# Serum microRNA microarray analysis identifies miR-4429 and miR-4689 are potential diagnostic biomarkers for biliary atresia

**DOI:** 10.1038/srep21084

**Published:** 2016-02-16

**Authors:** Rui Dong, Zhen Shen, Chao Zheng, Gong Chen, Shan Zheng

**Affiliations:** 1 Department of Pediatric Surgery, Children’s Hospital of Fudan University, and Key Laboratory of Neonatal Disease, Ministry of Health, 399 Wan Yuan Road, Shanghai 201102, China

## Abstract

This study aimed to investigate pathogenesis and novel diagnostic biomarkers of biliary atresia (BA). Serum samples from infants with BA and non-BA neonatal cholestasis (NC) were collected for miRNA microarray analysis, and then differentially expressed miRNAs were screened. Differentially expressed miRNAs were validated by qRT-PCR using an independent serum samples from infants with BA and NC. Diagnostic utility of validated miRNAs was further analyzed using serum samples by receiver-operating characteristic curve analysis. Totally, 13 differentially expressed miRNAs were identified including 11 down-regulated and 2 up-regulated ones. Target genes of hsa-miR-4429 and hsa-miR-4689 were significantly involved in FoxO signaling pathway. Eight differentially expressed miRNAs were chosen for validation by qRT-PCR analysis, and four miRNAs (hsa-miR-150-3p, hsa-miR-4429, hsa-miR-4689 and hsa-miR-92a-3p) were differentially expressed. The area under the curve of hsa-miR-4429 and hsa-miR-4689 was 0.789 (sensitivity = 83.33%, specificity = 80.00%) and 0.722 (sensitivity = 66.67%, specificity = 80.00%), respectively. Differentially expressed miRNAs including hsa-miR-4429 and hsa-miR-4689 might play critical roles in BA by regulating their target genes, and these two miRNAs may have the potential to become diagnostic biomarkers.

Biliary atresia (BA), a rare but serious cholestatic disorder in newborn infants, is caused by obstruction of extrahepatic or intrahepatic bile ducts with an occurrence rate of about 1/12,000 cases in United States and a higher incidence in Asia^1^,^2^. If not recognized and treated, BA will develop to progressive biliary cirrhosis and liver failure which could cause death within two years^3^. Kasai operation can reestablish bile flow in up to two-thirds of BA patients when performed earlier than 60 days of age^2^. However, a majority of patients will need liver transplant for survival if significant fibrosis and cirrhosis still exist after surgical intervention. Therefore, early identification and timely surgery are crucial for better prognosis. Unfortunately, definitive diagnosis of BA requires invasive and time-consuming diagnostic procedures, such as intra-operative cholangiogram and liver biopsy^4^. Consequently, it is urgently necessary for identification of noninvasive and convenient diagnostic biomarkers which may be helpful in distinguishing BA from the other neonatal cholestatic diseases.

MicroRNAs (miRNAs) are an abundant class of endogenous small and noncoding RNAs of about 22-nucleotides that post-transcriptionally regulate gene expressions^5^. Many studies have reported that miRNAs play important roles in experimental BA. The up-regulated expression of miR-29 in liver of murine BA model could lead to dysregulations of Igf1 and Il1RAP which are respectively responsible for cholangiocyte survival and modulation of inflammation^6^. In addition, miR-133a/b, miR-30b/c, miR-200a, miR-195, miR-365 and miR-320 have regulatory roles in pathogenesis of BA according to the miRNA expression profiles of extrahepatic bile ducts and gallbladder from murine BA model^7^. Shen *et al.* have reported that miR-222 was highly expressed in the extrahepatic bile ducts which might be responsible for liver fibrosis in the murine BA model^8^. The PI3K/Akt signaling pathway is activated in BA by elevated expression of miR-200b through suppressing FOG2, leading to increased growth and migration of human hepatic stallate cells^9^. Although understanding of BA pathogenesis has been improved, BA still remains to be a significant challenge and needs to be further investigated^10^. Therefore, it is of great importance to focus on the pathogenesis and diagnosis of BA.

Expression patterns of serum miRNAs can detect various diseases and distinguish similar disorders because serum levels of miRNAs are reproducible, stable and consistent among individuals of the same species^11^,^12^. What’s more, circulating miRNAs have been proposed as novel noninvasive biomarkers with encouraging diagnostic utility of BA. Zahm *et al.* have analyzed the serum miRNA expression profiles of BA patients and controls with indeterminate cholestasis by Low Density Array (TaqMan^®^ Array Human MicroRNA A Cards) and they found miR-200b/429 have promising diagnostic clinical performance for BA^13^. However, only 134 of 375 miRNAs were detected in the study of Zahm *et al.* and information about the molecular mechanisms of BA and diagnostic utility of serum miRNAs remains insufficient.

In order to screen more differentially expressed miRNAs, human microRNA microarrays from Agilent Technologies containing probes for 1523 miRNAs were adopted in this current study,. Meanwhile, advanced bioinformatics analysis including prediction of target genes, identification of interaction relationships between target genes and functional enrichment analysis was also conducted for better understanding of the molecular mechanisms of BA. Moreover, the differentially expressed miRNAs were further validated in a larger and independent cohort of 45 infants with BA and 30 controls with non-BA neonatal cholestasis to find potential serum miRNA biomarkers.

## Results

### Screening of differentially expressed miRNAs and clustering analysis

**Figure 1:**
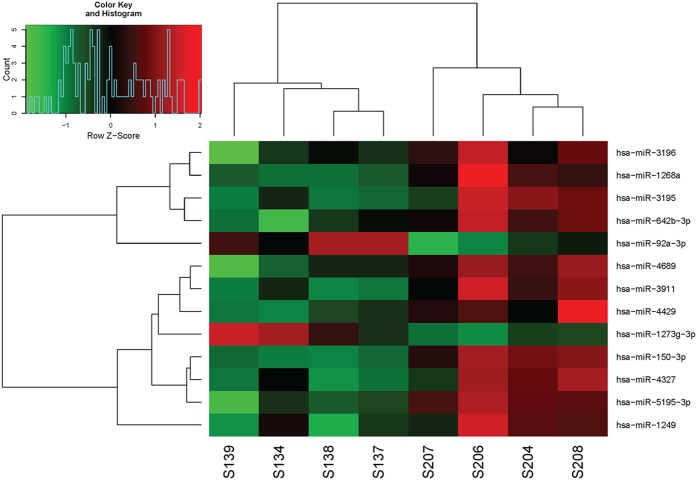
Hierarchical clustering analysis for the selected differentially expressed miRNAs. The horizontal axis represents the serum samples from infants with biliary atresia (BA) (S139, S134, S138 and S137) and non-BA neonatal cholestasis (NC) controls (S207, S206, S204 and S208). The miRNA names are shown on the left vertical axis. Colored bars indicate the range of fold changes.

The differentially expressed miRNAs between infants with BA and NC controls were screened using a human miRNA microarray. A total of 13 differentially expressed miRNAs were identified, including 11 down-regulated and 2 up-regulated miRNAs (Table 1). The serum expression levels of hsa-miR-1273 g-3p and hsa-miR-92a-3p were significantly increased in the infants with BA. Meanwhile, infants with BA had significantly lower expression levels of hsa-miR-1268a, hsa-miR-3911, hsa-miR-4689, hsa-miR-3196, hsa-miR-4429, hsa-miR-4327, hsa-miR-150-3p, hsa-miR-642b-3p, hsa-miR-1249, hsa-miR-3195 and hsa-miR-5195-3p. Furthermore, hierarchical clustering analysis indicated the serum samples of infants with BA could be well distinguished from NC controls according to the expression levels of these 13 differentially expressed miRNAs (Fig. 1).

### Functional and pathway enrichment analysis

The number of predicted target genes for hsa-miR-1268a, hsa-miR-3911, hsa-miR-4689, hsa-miR-3196, hsa-miR-4429, hsa-miR-4327, hsa-miR-150-3p, hsa-miR-642b-3p, hsa-miR-1249, hsa-miR-3195, hsa-miR-5195-3p, hsa-miR-92a-3p and hsa-miR-1273 g-3p was 15, 26, 24, 35, 182, 131, 115, 333, 9, 9, 183, 209 and 74, respectively (Supplementary Table 1).

The functional enrichment analysis revealed that target genes of hsa-miR-150-3p (FOXO3 and SMAD5), hsa-miR-642b-3p (WT1, PROX1, NFYB, CBL and RB1), hsa-miR-4327 (CREBBP, SALL1 and SOX2), hsa-miR-5195-3p (SMAD3, SOX9 and CITED2), hsa-miR-4429 (CREB5 and TFAP2B) and hsa-miR-92a-3p (RNF4, SOX4 and HNF1B) were significantly related to the protein binding (FDR = 0.000126268), sequence-specific DNA binding transcription factor activity (FDR = 0.001452774), positive regulation of transcription, DNA-dependent (FDR = 0.004632587) and nucleus (FDR = 0.00852864) (Table 2). The ROCK1 and ANK3 respectively targeted by hsa-miR-4689 and hsa-miR-3911 were also involved with protein binding. Another target gene of hsa-miR-4689 (SGK1) and hsa-miR-3911 (PLCB1) were related to nucleus. The members of frizzled (FZD) family (FZD1, FZD2 and FZD3) and G protein-coupled receptors (such as GPR64 targeted by hsa-miR-1249) were related to G-protein coupled receptor activity (FDR = 0.000125237). Meanwhile, FZD1 and FZD2 which were respectively targeted by hsa-miR-642b-3p and hsa-miR-3196 were also involved with the positive regulation of transcription, DNA-dependent.

There were 29 significantly enriched pathways for the target genes (Supplementary Table 2), and the top ten pathways are listed in the Table 2. The target genes of hsa-miR-642b-3p (such as PPM1A, PAK2, PTPRR, PPKACB and MAP2K6), hsa-miR-4429 (such as MAPK1, AKT3, CACNG2, PPM1B, RAP1A and RASA1), hsa-miR-92a-3p (such as RAP1B, MAP2K4, NLK, DUSP10 and CACNA1I) and hsa-miR-3196 (such as JUND) were significantly enriched in the MAPK signaling pathway (FDR = 0.016391713, Table 3). Meanwhile, MAPK1, AKT3 and IGF1R regulated by hsa-miR-4429 also participate in the Proteoglycans in cancer (FDR = 0.004268857), Rap1 signaling pathway (FDR = 0.018406673) and Ras signaling pathway (FDR = 0.024181237). Besides, ROCK1 targeted by hsa-miR-4689 and PLCB1 targeted by hsa-miR-3911 were significantly involved in Proteoglycans in cancer, and PLCB1 was Rap1 signaling pathway and Glutamatergic synapse (FDR = 0.022324586). Moreover, the target genes of hsa-miR-4327 (CREBBP and IGF1R), hsa-miR-4689 (SGK1), hsa-miR-150-3p (IGF1, PRKAA2 and FOXO3), hsa-miR-4429 (MAPK1, AKT3, IGF1R and PTEN) and hsa-miR-92a-3p (NLK, KLF2 and PTEN) were revealed to participate in FoxO signaling pathway (FDR = 0.036456906).

**Figure 2:**
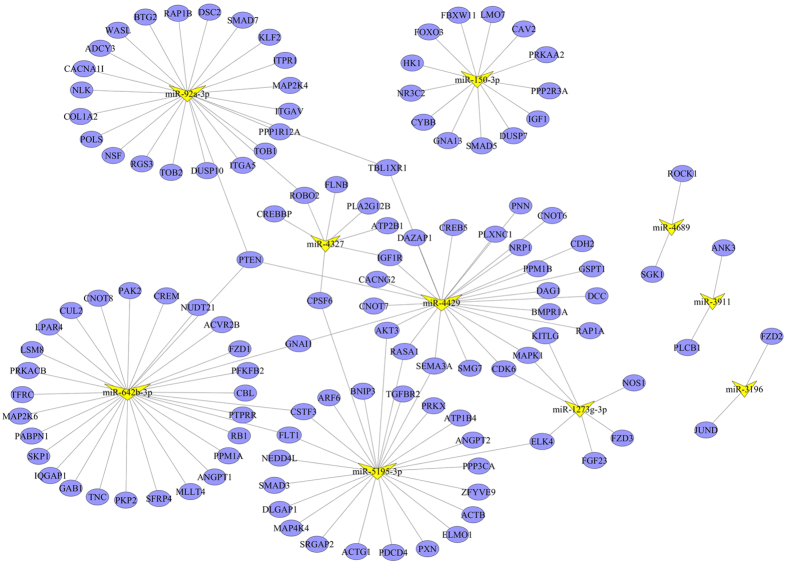
The regulatory network for differentially expressed miRNAs. The blue nodes represent the target genes. The yellow triangles indicate differentially expressed miRNAs with the size corresponding to degree. The blue lines show the potential regulatory relationships between miRNAs and genes.

**Figure 3:**
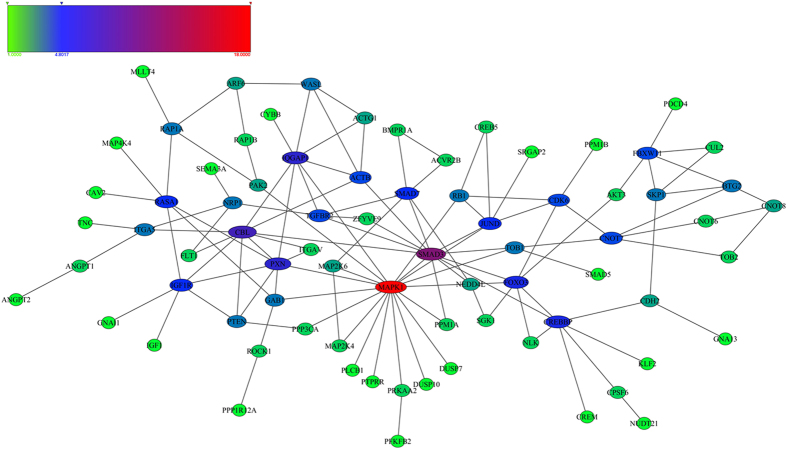
Protein-protein interaction (PPI) network for the predicted target genes of differentially expressed miRNAs. Colored bars indicate the degree of genes.

In the constructed regulatory network (Fig. 2), PTEN was simultaneously targeted by hsa-miR-92a-3p, hsa-miR-4429 and hsa-miR-642b-3p. The MAPK1 had the highest degree of 18 in the PPI network, followed by SMAD3 (degree = 12) and CBL (degree = 9) (Fig. 3, Table 4). Moreover, PTEN was interacted with IGF1R, PXN, CBL and PPP3CA in the PPI network.

### Validation of important miRNAs and enrichment analysis

**Figure 4:**
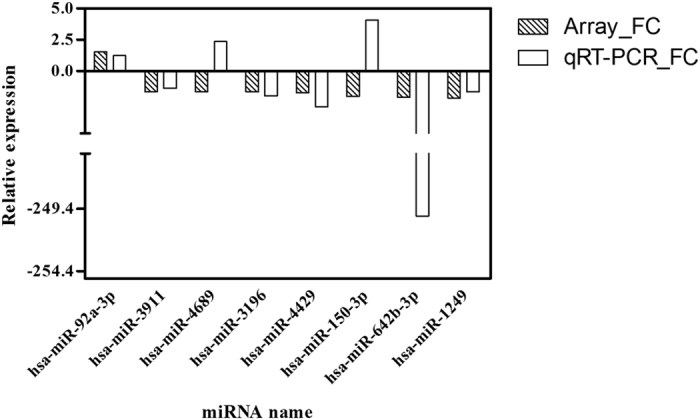
Validation of selected miRNAs by qRT-PCR. Serum expression levels of hsa-miR-92a-3p, hsa-miR-3911, hsa-miR-4689, hsa-miR-3196, hsa-miR-4429, hsa-miR-150-3p, hsa-miR-642b-3p and hsa-miR-1249 were measured in 10 infants with BA and 10 NC controls.

Eight differentially expressed miRNAs (hsa-miR-92a-3p, hsa-miR-3911, hsa-miR-4689, hsa-miR-3196, hsa-miR-4429, hsa-miR-150-3p, hsa-miR-642b-3p and hsa-miR-1249) whose target genes were significantly enriched in important functions and pathways were selected as putative biomarkers for further validation. The expression levels of these eight candidate miRNAs were firstly measured by qRT-PCR in an independent cohort of serum samples from 10 infants with BA and 10 NC controls. The qRT-PCR revealed that hsa-miR-92a-3p and hsa-miR-4429 were respectively up-regulated and down-regulated with significant differences, showed the same change patterns as shown in with microarray analysis. However, the expression levels of hsa-miR-4689 (fold change = 2.36) and hsa-miR-150-3p (fold change = 4.08) were significantly increased which were opposite to the microarray analysis (Fig. 4). This discrepancy between first screening and validation might be due to technical limitations of microarray, such as cross-hybridizations, signal saturations and limited dynamic range^14^. The other four miRNAs were excluded for further analysis since no significant differences were detected.

The functional enrichment analysis indicated that target genes of hsa-miR-150-3p (FOXO3 and SMAD5), hsa-miR-4429 (CREB5 and TFAP2B), hsa-miR-4689 (ROCK1) and hsa-miR-92a-3p (RNF4, SOX4 and HNF1B) were significantly related to the protein binding (FDR = 0.015416) (Table 5). The GNAI1 targeted by hsa-miR-4429 and GPR180 by hsa-miR-92a-3p were significantly related to the function of G-protein coupled receptor signaling pathway (FDR = 4.92E-05). A totoal of 14 significant pathways were enriched for the target genes of hsa-miR-150-3p, hsa-miR-4429, hsa-miR-4689 and hsa-miR-92a-3p (Table 6). There were 12 target genes (AKT3, CPD, FOXO3, IGF1, IGF1R, KLF2, MAPK1, NLK, NRAS, PRKAA2, PTEN and SGK1) that revealed to participate in FoxO signaling pathway (FDR = 0.007496). Meanwhile, target genes of hsa-miR-150-3p (IGF1 and PRKAA2), hsa-miR-4429 (AKT3, MAPK1, PTEN and TSC1), hsa-miR-92a-3p (TSC1 and PTEN) were simultaneously involved in the mTOR signaling pathway (FDR = 0.012814) and PI3K-Akt signaling pathway (FDR = 0.020308).

### Diagnostic utility of potential miRNAs

**Figure 5:**
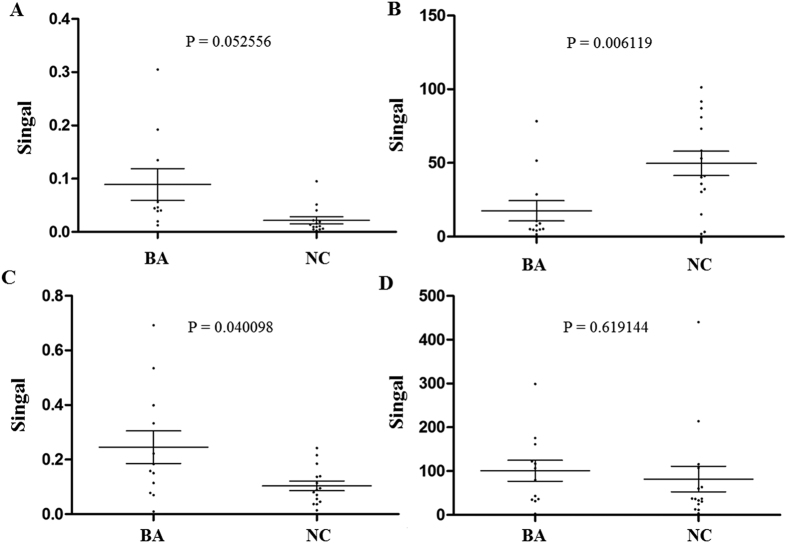
The expression levels of hsa-miR-150-3p, hsa-miR-4429, hsa-miR-4689 and hsa-miR-92a-3p in an independent set of serum samples from infants with BA (n = 35) and NC controls (n = 20) (**A**) hsa-miR-150-3p; (**B**) hsa-miR-4429; (**C**) hsa-miR-4689; (**D**) hsa-miR-92a-3p).

**Figure 6:**
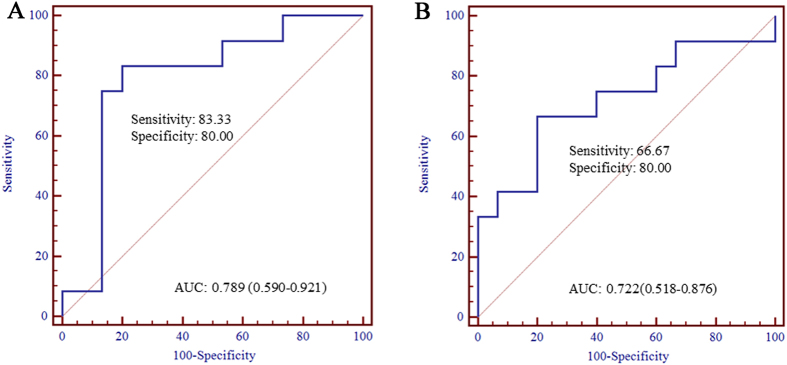
Receiver operating characteristic curve analysis for BA diagnosis (**A**) hsa-miR-4429, (**B**) hsa-miR-4689; AUC: area under the curve).

The hsa-miR-150-3p, hsa-miR-4429, hsa-miR-4689 and hsa-miR-92a-3p were selected for subsequent analysis of diagnostic utility. The expression levels of these miRNAs were detected by qRT-PCR using serum samples from another 35 infants with BA and 20 NC controls. Significantly down-regulated expression levels of hsa-miR-4429 were observed in serum samples of infants with BA in comparison to NC controls (P = 0.006119, Fig. 5B). The serum expression levels of hsa-miR-4689 were obviously higher in infants with BA (P = 0.040098, Fig. 5C). However, there was no significant differences in the expression levels of hsa-miR-150-3p (P = 0.052556) and hsa-miR-92a-3p (P = 0.619144) (Fig. 5A,D). After constructing ROC curves, the cutoff value for hsa-miR-4429 was 28.7112, with a sensitivity of 83.33% and a specificity of 80.00%. At the cutoff value of 0.1388 for hsa-miR-4689, the sensitivity was 66.67% and the specificity was 80.00%. The AUC was 0.789 (95% CI, 0.590–0.921) and 0.722 (95% CI, 0.518–0.876) for hsa-miR-4429 and hsa-miR-4689, respectively (Fig. 6).

## Discussion

Long-term survival can be achieved without liver transplantation when the Kasai procedure is conducted within the first 30–45 days of life^15^. Since early diagnosis and convenient screening means of BA haven’t been well established, treatment for BA is inadequate due to delayed diagnosis and poor understanding of the pathogenesis. In this study, 13 differentially expressed miRNAs were identified using the Agilent miRNA microarray expression profiling. Moreover, eight differentially expressed miRNAs were selected for validation by qRT-PCR using an independent serum samples from infants with BA and NC controls. The validation analysis revealed that four miRNAs including hsa-miR-150-3p, hsa-miR-4429, hsa-miR-4689 and hsa-miR-92a-3p were differentially expressed.

In our study, functional enrichment analysis indicated that GNAI1 targeted by hsa-miR-4429 and GPR180 by hsa-miR-92a-3p were significantly related to the function of G-protein coupled receptor signaling pathway. It has been revealed that secretin can contribute to increased ductular choleresis after stimulation of G-protein-coupled receptor and cAMP/protein kinase A (PKA)-dependent signalling pathway^16^. Moreover, decreased expression of the type III InsP3R in the cholangiocytes of patients with biliary atresia is associated with impaired Ca2+ signaling and messenger molecule interacts with specific G protein-coupled receptors can induce activation of phospholipase C and formation of InsP3 to increase Ca^2^+ 17,18. Accordingly, hsa-miR-4429 and hsa-miR-92a-3p may be important for BA by targeting GNAI1 and GPR180 to influence the G-protein coupled receptor signaling pathway.

Moreover, target genes of hsa-miR-150-3p (IGF1, PRKAA2 and FOXO3), hsa-miR-4689 (SGK1), hsa-miR-4429 (MAPK1, AKT3, IGF1R and PTEN) and hsa-miR-92a-3p (NLK, KLF2 and PTEN) were significantly enriched in FoxO signaling pathway. BA is manifested by progressive inflammation and fibrosis of extrahepatic and intrahepatic bile ducts which can lead to cirrhosis^19^. It has been reported the FoxO3/Bim signaling pathway was obviously activated in patients with primary biliary cirrhosis^20^. Meanwhile, target genes of hsa-miR-4429 (AKT3, MAPK1, PTEN and TSC1) were significantly involved in the mTOR and PI3K-Akt signaling pathway. Increased expression levels of miR-200b in biliary atresia patients can acitvate PI3K/Akt signaling to accelerate migration and proliferation of hepatic stallate cells^9^. It has been reported that cystic proliferation of cholangiocytes of the polycystic kidney rat is associated with activation of the PI3K/mTOR pathway^21^. Therefore, it could be speculated thathsa-miR-4429 and hsa-miR-4689 might play important roles in BA by regulating their target genes that participate in these important signaling pathways.

Furthermore, the diagnostic utility of these four miRNAs (hsa-miR-150-3p, hsa-miR-4429, hsa-miR-4689 and hsa-miR-92a-3p) were analyzed in a larger independent sample set from another 35 infants with BA and 20 NC controls. The AUC of hsa-miR-4429 and hsa-miR-4689 was respectively 0.789 (95% CI, 0.590–0.921; sensitivity = 83.33%, specificity = 80.00%) and 0.722 (95% CI, 0.518–0.876; sensitivity = 66.67%, specificity = 80.00%), suggesting hsa-miR-4429 and hsa-miR-4689 might have the potential to be used as diagnostic biomarkers of BA. The human serum samples in the study of Zahm *et al.* were obtained from the Childhood Liver Disease Research and Education Network’s prospective longitudinal study of cholestasis in infancy, and the results revealed that miR-200b/429 might has promising diagnostic performance with the AUC values greater than 0.80^13^. However, the expression levels of miR-200b/429 didn’t significantly altered in the serum samples of infants with type III BA aged less than 90 days enrolled from the Children’s Hospital of Fudan University in our study. It could be speculated the different results might be due to that samples obtained from different sources were investigated. However, there are some limitations in our study. Some characteristics of participates, such as ethnic and region, were not considered due to the limited sample size. Besides, the expression levels of import target genes were not studied.

In conclusion, the differentially expressed miRNAs (especially hsa-miR-150-3p, hsa-miR-4429, hsa-miR-4689 and hsa-miR-92a-3p) might play important roles in the pathogenesis of BA by regulating their target genes. Furthermore, hsa-miR-4429 and hsa-miR-4689 might have promising diagnostic performance for BA. In our next study, expression levels of important target genes will be investigated by integrated miRNA and mRNA analysis to find more potential biomarkers for BA.

## Methods

### Study population and sample collection

In our study, 45 infants with BA and 30 infants with non-BA neonatal cholestasis (NC) similar in age and sex distribution during the same time period were enrolled from the Children’s Hospital of Fudan University (Table 7). These participants were enrolled based on the diagnoses of operative cholangiogram and liver pathology. The inclusion criteria for infants with BA were as follows: aged less than 90 days; type III BA according to the classification of BA phenotype^22^; serum direct or conjugated hyperbilirubinemia (>20% of total bilirubin and >2 mg/dL). Infants were excluded when they had liver failure, malignancy, ischemic hepatopathy, hypoxia or shock within the preceding 2 weeks; treated with extracorporeal membrane oxygenation–associated cholestasis or prior hepatobiliary surgery. Meanwhile, infants with birth weight less than 1500 g, drug- or total parenteral nutrition-associated cholestasis, bacterial or fungal sepsis, or primary hemolytic disease were also excluded unless they were diagnosed with BA or another cholestatic disease definitively^23^,^24^.

Blood samples were collected from these participants within a few days of enrollment preoperatively when the parents had given voluntary informed consent for their children. Our studies have been reviewed and approved by the ethics committee of the Children’s Hospital of Fudan University and all the experiments were carried out in accordance with relevant guidelines and regulations.

### Sample processing and total RNA isolation

All whole blood samples collected from each participant were allowed to stand for about 1 h at room temperature. Then, these whole blood samples were separated into serum by centrifugation at 820 × g for 10 min at 4 °C, followed by further centrifugation at 16,000 × g for 10 min at 4 °C to completely remove cell debris. The supernatant serum was stored at −20 °C until analysis. Total RNA was isolated from 400 μl serum sample by using mirVana miRNA isolation kit (Applied Biosystems, Foster City, CA) according to the manufacturer’s instructions. The concentration and quality of total RNA were monitored by NanoDrop ND-2000 spectrophotometer (Thermo Fisher Scientific, Waltham, MA) and Agilent’s 2100 Bioanalyzer (Agilent Technologies, Santa Clara, CA).

### Serum miRNA expression profiling and microarray analysis

Serum samples of 4 infants with BA and 4 NC controls were used for miRNA microarray analysis. Human miRNA microarrays from Agilent Technologies (8*60 K), containing probes for 1523 human miRNAs from the Sanger miRbase V18.0 database (http://www.sanger.ac.uk/Software/ Rfam/ mirna), were adopted. Total RNA (100 ng) extracted from each serum sample was used as inputs for sample labeling and hybridization preparation in accordance with the manufacturer’s protocol (Agilent Technologies, Santa Clara, CA).

The microarray image information was converted into spot intensity values using Scanner Control Software Rev. 7.0 (Agilent Technologies, Santa Clara, CA). The signal after background subtraction was exported directly into the GeneSpring GX version 12.5 software (Agilent Technologies, Santa Clara, CA) for quartile normalization. Then, differentially expressed miRNAs in serum were identified using the paired *t*-test with the cut-off criteria of P < 0.05 and |fold change|>1.5. In order to ensure the screened differentially expressed miRNAs were accurately identified, hierarchical clustering analysis of samples was employed using heatmap.2 function of th gplots package in R^25^ based on the expression values.

The target genes of differentially expressed miRNAs were predicted by at least two databases of the following five usual prediction databases: TargetScan (http://www.targetscan.org), miRanda (http://www.microrna.org/microrna/home.do), PicTar (http://pictar.mdc-berlin.de/), MirTarget2 from miRDB (http://mirdb.org/miRDB/download.html) and PITA (http://genie.weizmann.ac.il/pubs/mir07/mir07_prediction.html). Moreover, the Gene Ontology (GO) functional and pathway enrichment analysis were conducted for the target genes using the Database for Annotation, Visualization and Integrated Discovery (DAVID) online tools^26^ with the cut-off criterion of false discovery rate (FDR) < 0.05. The GO terms were identified in biological process (BP), cellular component (CC) and molecular function (MF) categories. The regulatory relationships for targets genes that simultaneously involved in significantly enriched functions and pathways were selected to constructe miRNA-target gene regulatory network. Protein-protein interactions (PPIs) for these target genes were revealed by the genemania (http://www.genemania.org/). The miRNA-target gene regulatory network and PPI network were both visualized using Cytoscape (Version 3.1.1)^27^.

### Quantitative real-time PCR (qRT-PCR)

The relative quantification of selected differentially expressed miRNAs was performed by qRT-PCR reaction with the miScript SYBR Green PCR Kit (Qiagen) using ABI 7500 Real-Time PCR System (Applied Biosystems, Foster City, CA, USA). The miRNA specific primers were designed by Primer Express software (Version 2.0, Applied Biosystems) based on the miRNA sequences obtained from miRbase database (http://microrna.sanger.ac.uk/). Primer sequences are listed in the Table 8.

Extracted total RNA (60 ng) from serum samples was reverse transcribed into cDNA using miScript Reverse Transcription Kit (Qiagen). Each reaction was performed in a 20 μl volume system containing 1.5 μl cDNA, 2 μl of each primer and 1 × QuantiTect SYBR Green PCR Master Mix (Qiagen). MiR-1228 was used as a stable endogenous control for normalization since it functions as a housekeeping gene according to the study of Hu *et al.*^28^. All reactions were carried out in triplicate. The relative expression levels of miRNAs were calculated by the 2^−△△Ct^ method.

### Statistics

Receiver-operating characteristic (ROC) curve analysis was performed to determine the specificity and sensitivity of miRNA as a diagnostic biomarker. MedCalc (version 10.4.7.0; MedCalc, Mariakerke, Belgium) software was adopted to perform ROC analysis. Area under the ROC curve (AUC) was calculated as an accuracy index for evaluating the diagnostic performance of selected miRNA. The 95% confidence interval (CI) was used to reflect statistical significance.

## Additional Information

**How to cite this article**: Dong, R. *et al.* Serum microRNA microarray analysis identifies miR-4429 and miR-4689 are potential diagnostic biomarkers for biliary atresia. *Sci. Rep.* **6**, 21084; doi: 10.1038/srep21084 (2016).

## Supplementary Material

Supplementary Information

## Figures and Tables

**Table 1:** The screened differentially expressed miRNAs in the serum samples of infants with biliary atresia.

MiRNAs	Name	Fold change	P value
Down-regulated	hsa-miR-1268a	−1.52824	0.031041
	hsa-miR-3911	−1.64985	0.014516
	hsa-miR-4689	−1.65676	0.015478
	hsa-miR-3196	−1.68217	0.043657
	hsa-miR-4429	−1.72581	0.042748
	hsa-miR-4327	−1.75703	0.026081
	hsa-miR-150-3p	−2.03072	0.002125
	hsa-miR-642b-3p	−2.10194	0.01667
	hsa-miR-1249	−2.16191	0.039503
	hsa-miR-3195	−2.23659	0.034854
	hsa-miR-5195-3p	−2.45733	0.005206
Up-regulated	hsa-miR-92a-3p	1.528466	0.011198
	hsa-miR-1273g-3p	2.342245	0.039625

**Table 2:** The enriched Gene Ontology (GO) terms in molecular function (MF), biological process (BP) and cellular component (CC) categories for target genes of all the 13 differentially expressed miRNAs.

GO_ID	GO_term	Category	Count	Target Genes	FDR
GO:0005515	Protein binding	MF	433	FZD1, FZD3, IGF1R, CBL, CREBBP, FOXO3, SMAD3, SMAD5, RB1, SALL1, SOX2, SOX4, TFAP2B, WT1, CREB5	0.000126268
GO:0004930	G-protein coupled receptor activity	MF	11	FZD2, LPAR4, GPR34, FZD3, FZD1, GPR64, GPR75, GPR126, ELTD1, GPR115, TAS2R20	0.000125237
GO:0003700	Sequence-specific DNA binding transcription factor activity	MF	100	CBL, CREBBP, CREM, ELK4, FOXO3, GABPB1, SMAD3, SMAD5, SMAD7, PROX1, RB1, SALL1, SOX2, SOX4,TFAP2B,WT1, CREB5…	0.001452774
GO:0001105	RNA polymerase II transcription coactivator activity	MF	8	CREBBP, POU3F1, SOX4, SOX11, TFAP2B, CITED2, ANKRD1, HIPK2	0.049058312
GO:0045893	Positive regulation of transcription, DNA-dependent	BP	61	FZD1, FZD2, CREBBP, SMAD3, SMAD5, NFYB, PROX1, RB1, RNF4, SALL1, SOX2, SOX4, SOX9, HNF1B, TFAP2B, WT1, CREB5, CITED2	0.004632587
GO:0006351	Transcription, DNA-dependent	BP	170	CREM, ELK4, GABPB1, GATA2, HIC1, HOXA5, JUND, SMAD3, SMAD5, SMAD7, MTF1, NFYB, PBX1, RB1, RNF4, SALL1, SOX2	0.004014426
GO:0045944	Positive regulation of transcription from RNA polymerase II promoter	BP	77	JUND, SMAD3, SMAD5, SMAD7, MTF1, PBX1, PROX1, RB1, RNF4, SALL1, SOX2, SOX4, SOX9, HNF1B, TFAP2B,	0.006415167
GO:0007156	Homophilic cell adhesion	BP	25	CDH2, CDH10, DSC2, PCDH7, ROBO2, MPZL2, NPTN, PCDH11X, CDH20, PCDH18	0.008450129
GO:0005634	Nucleus	CC	396	CBL, CREBBP, SMAD3, SMAD5, RB1, RNF4, SALL1, SOX2, SOX9, HNF1B, ZEB1, TFAP2B, WT1, CREB5,	0.00852864
GO:0005576	Extracellular region	CC	56	ADM, ANGPT2, COL1A2, EREG, FBN1, IGF1, IGSF1, KITLG, NOTCH3, YBX1, LRP8, ADAM12, NRP1, CD200R1	0.009038887
GO:0005883	Neurofilament	CC	6	NEFM, NEFH, NEFL, NRP1, INA, DLGAP2	0.017283848

FDR: false discovery rate.

**Table 3:** The top ten enriched pathways for target genes of all the 13 differentially expressed miRNAs.

Pathway_ID	Name	Count	Target Genes	FDR
hsa04740	Olfactory transduction	5	CALM1, ADCY3, PRKX, PRKACB, ADRBK2	0.001989049
hsa05205	Proteoglycans in cancer	31	ITGAV, ITGA5, IGF1, FRS2, CAV2, PIK3R3, MAPK1, PLCE1, GAB1, ITPR1, AKT3, ACTB, IGF1R, IQGAP1, FZD3, PRKACB, ROCK1	0.004268857
hsa04010	MAPK signaling pathway	32	PPM1A, RAP1B, MAPK1, CACNG2, MAP4K4, AKT3, NLK, DUSP10, PTPRR, RASA1, MAP2K4, PPM1B, RAP1A, PRKACB, CACNA1I, MAP2K6	0.016391713
hsa05414	Dilated cardiomyopathy	15	ACTG1, ITGB8, ITGAV, DAG1, ITGA5, IGF1, CACNA1D, ATP2A2, CACNG2, ADCY3, ACTB, PRKX, CACNA2D4, PRKACB, TPM3	0.02136154
hsa04015	Rap1 signaling pathway	27	CNR1, MLLT4, FLT1, IGF1, GNAI1, CALM1, RAP1B, ANGPT1, PIK3R3, MAPK1, PLCE1, LPAR4, AKT3, ADCY3, ACTB, IGF1R	0.018406673
hsa04520	Adherens junction	13	ACTG1, CREBBP, MLLT4, YES1, LMO7, WASL, MAPK1, NLK, ACTB, IGF1R, SMAD3, TGFBR2, IQGAP1	0.016409495
hsa05412	Arrhythmogenic right ventricular cardiomyopathy (ARVC)	13	ACTG1, CDH2, ITGB8, ITGAV, DAG1, ITGA5, DSC2, CACNA1D, PKP2, ATP2A2, CACNG2, ACTB, CACNA2D4	0.016093657
hsa04724	Glutamatergic synapse	17	GRIK5, GNAI1, CACNA1D, SLC1A2, MAPK1, ITPR1, PPP3CA, ADCY3, SLC17A6, DLGAP1, GNG7, PRKX	0.022324586
hsa04360	Axon guidance	18	NRAS, DCC, SEMA6D, SRGAP1, SEMA3A, SEMA6A, PAK7, GNAI1, NRP1, MAPK1, ROBO2, PAK2	0.02165145
hsa04014	Ras signaling pathway	27	IGF1, CALM1, RAP1B, ANGPT1, PIK3R3, MAPK1, PLCE1, ARF6, GAB1, AKT3, IGF1R, PAK2, FGF23, GNG7	0.024181237

**Table 4:** The target genes with degrees not less than five in the protein-protein interaction network.

Target genes	Degree
MAPK1	18
SMAD3	12
CBL	9
PXN	8
IQGAP1	8
FOXO3	7
CREBBP	7
SMAD7	6
JUND	6
RASA1	6
IGF1R	6
CNOT7	5
FBXW11	5
ACTB	5
TGFBR2	5
CDK6	5

**Table 5:** The enriched GO terms for target genes of hsa-miR-150-3p, hsa-miR-4429, hsa-miR-4689 and hsa-miR-92a-3p.

GO_ID	GO_term	Category	Count	Target Genes	FDR
GO:0001077	RNA polymerase II core promoter proximal region sequence-specific DNA binding transcription factor activity involved in positive regulation of transcription	MF	15	KLF5, GATA2, MYB, NFIA, PBX1, SOX4, TCF21, TFAP2B, BTG2, KLF4, ONECUT2, CREB5, EHF, RIT1, MYOCD	0.012047
GO:0005515	Protein binding	MF	232	FOXO3, SMAD5, CREB5, KLF5, RNF4, HNF1B, ROCK1, GATA2, MYB, PBX1, SOX4, TFAP2B, BTG2, KLF4, CREB5, RIT1, MYOCD	0.015416
GO:0007156	Homophilic cell adhesion via plasma membrane adhesion molecules	BP	22	CDH2, CDH10, DSC2, ROBO2, NPTN, PCDH11X, CDH20, PCDHAC2, PCDHAC1, PCDHA13, PCDHA12, PCDH19, PCDH11Y…	8.62E-07
GO:0007186	G-protein coupled receptor signaling pathway	BP	2	GNAI1, GPR180	4.92E-05
GO:0045944	Positive regulation of transcription from RNA polymerase II promoter	BP	50	ATRX, KLF5, CCNC, CUX1, DDX3X, ESRRG, FOXO3, GATA2, HOXA5, IGF1, SMAD7, PPP1R12A, NFIA, NPAS2, PAX9, RNF4, SOX4, DHX36…	0.00105
GO:0017148	Negative regulation of translation	BP	9	DDX3X, TSC1, BTG2, FXR1, TOB1, SYNCRIP, IGF2BP3, CPEB3, NANOS1	0.002412
GO:0033693	Neurofilament bundle assembly	BP	3	NEFM, NEFH, NEFL	0.012468
GO:0018107	Peptidyl-threonine phosphorylation	BP	7	DYRK1A, MAPK1, TNKS, OXSR1, HIPK3, NLK, WNK1	0.014844
GO:0005883	Neurofilament	CC	6	NEFM, NEFH, NEFL, NRP1, INA, DLGAP2	0.000132
GO:0005730	Nucleolus	CC	74	HNF1B, TDG, TEAD1, NR2C2, FXR1, DYRK2, CGGBP1, TNKS, BAZ2A, DUSP10, RTF1, DAZAP1, HBP1, AFF4, GRHL1, ERGIC2, WAC, POLK	0.010514

**Table 6:** The enriched pathways for target genes of hsa-miR-150-3p, hsa-miR-4429, hsa-miR-4689 and hsa-miR-92a-3p.

Pathway_ID	Name	Count	Target genes	FDR
hsa03018	RNA degradation	10	BTG2, CNOT6, CNOT7, DHX36, PAN3, PAPD5, POLS, TOB1, TOB2, XRN1	0.00254
hsa04730	Long-term depression	8	GNA13, GNAI1, GRIA3, IGF1, IGF1R, ITPR1, MAPK1, NRAS	0.007796
hsa04360	Axon guidance	12	DCC, GNAI1, MAPK1, NRAS, NRP1, PAK7, PLXNC1, RGS3, ROBO2, ROCK1, SEMA3A, SEMA6D	0.006358
hsa04068	FoxO signaling pathway	12	AKT3, CPD, FOXO3, IGF1, IGF1R, KLF2, MAPK1, NLK, NRAS, PRKAA2, PTEN, SGK1	0.007496
hsa05410	Hypertrophic cardiomyopathy (HCM)	9	ATP2A2, CACNA2D4, CACNG2, DAG1, IGF1, ITGA5, ITGAV, PRKAA2, TPM3	0.007272
hsa05414	Dilated cardiomyopathy	9	ADCY3, ATP2A2, CACNA2D4, CACNG2, DAG1, IGF1, ITGA5, ITGAV, TPM3	0.011524
hsa04150	mTOR signaling pathway	7	AKT3, IGF1, MAPK1, PRKAA2, PTEN, TSC1, ULK1	0.012814
hsa04611	Platelet activation	11	ADCY3, AKT3, COL1A2, GNA13, GNAI1, ITPR1, MAPK1, PPP1R12A, RAP1A, RAP1B, ROCK1	0.012208
hsa05214	Glioma	7	AKT3, CDK6, IGF1, IGF1R, MAPK1, NRAS, PTEN	0.016841
hsa04510	Focal adhesion	14	AKT3, CAV2, COL1A2, IGF1, IGF1R, ITGA5, ITGAV, MAPK1, PAK7, PPP1R12A, PTEN, RAP1A, RAP1B, ROCK1	0.02147
hsa04151	PI3K-Akt signaling pathway	20	AKT3, CDK6, COL1A2, CREB5, FOXO3, IGF1, IGF1R, ITGA5, ITGAV, KITLG, MAPK1, MCL1, MYB, NRAS, PHLPPL, PPP2R3A, PRKAA2, PTEN, SGK1, TSC1	0.020308
hsa05218	Melanoma	7	AKT3, CDK6, IGF1, IGF1R, MAPK1, NRAS, PTEN	0.022286
hsa03015	mRNA surveillance pathway	8	DAZAP1, GSPT1, HBS1L, MSI2, NXT2, PNN, PPP2R3A, SMG7	0.024566
hsa05412	Arrhythmogenic right ventricular cardiomyopathy (ARVC)	7	ATP2A2, CACNA2D4, CACNG2, CDH2, DAG1, ITGA5, ITGAV	0.024824

**Table 7:** Distribution of study subjects and liver function tests.

	BA	NC	P value
Age (days)*	70.30 ± 15.15	64.25 ± 10.33	0.32
Male/Female	25/20	18/12	0.35
Diagnosis type	III^1^		N/A
TB (μmol/L)	188.52 ± 100.36	158.62 ± 50.46	0.59
DB (μmol/L)	150.45 ± 71.25	125.32 ± 35.33	0.51
DB/TB	0.76 ± 0.45	0.74 ± 0.36	0.11
AST (IU/L)	268.15 ± 220.40	225.50 ± 200.67	0.15
ALT (IU/L)	135.10 ± 105.25	146.30 ± 135.25	0.55
γ-GGT	766.35 ± 650.67	245.15 ± 210.42	< 0.01

Type III biliary atresia refers to the discontinuity of both right and left hepatic ducts to the level of porta hepatis. Unfortunately, type III BA is common, accounting for >90% of cases. *at liver biopsy sample day.

BA: biliary atresia; NC: non-BA neonatal cholestasis infants; ALT: Alanine transaminase; AST: Aspartate transaminase; DB: Direct bilirubin; TB: Total bilirubin; γ-GGT: Gamma glutamyl transpeptidase.

**Table 8:** Primers used for quantitative real-time PCR (qRT-PCR).

miRNAs	Primer sequences
hsa-miR-92a-3p	UAUUGCACUUGUCCCGGCCUGU
hsa-miR-3911	UGUGUGGAUCCUGGAGGAGGC
hsa-miR-4689	UUGAGGAGACAUGGUGGGGGCC
hsa-miR-3196	CGGGGCGGCAGGGGCCU
hsa-miR-4429	AAAAGCUGGGCUGAGAGGCG
hsa-miR-150-3p	CUGGUACAGGCCUGGGGGACAG
hsa-miR-642b-3p	AGACACAUUUGGAGAGGGACCC
hsa-miR-1249	ACGCCCUUCCCCCCCUUCUUCA
has-miR-1228	UCACACCUGCCUCGCCCCCC
