# Supplementary material for: Serum microRNA microarray analysis identifies miR-4429 and miR-4689 are potential diagnostic biomarkers for biliary atresia
Source: Sci Rep. 2016 Feb 16;6:21084. doi: 10.1038/srep21084 (PMC4754688; doi:10.1038/srep21084)
Supplement: Supplementary Information [file srep21084-s1.doc]

**Serum microRNA microarray analysis identifies miR-4429 and miR-4689 are potential diagnostic biomarkers for biliary atresia**

Rui Dong^1, +^, Zhen Shen^1, +^, Chao Zheng^1^, Gong Chen^1^, Shan Zheng ^1, *^

^1^Department of Pediatric Surgery, Children’s Hospital of Fudan University, and Key Laboratory of Neonatal Disease, Ministry of Health, 399 Wan Yuan Road, Shanghai 201102, China

^*^Correspondence: Shan Zheng, Fax: +86 021 64931901; Tel: +86 021 64931007. E-mail address: [szheng@shmu.edu.cn](mailto:szheng@shmu.edu.cn)

^+^ These authors contributed equally to this manuscript and should be considered as co-first authors

**Supplementary Table 1.** Target genes of the differentially expressed miRNAs

| MiRNAs | Number | Target genes |
| --- | --- | --- |
| hsa-miR-1268a | 15 | KCNA3, EGR4, TMEM229A, TARP, ZFP41, PATE1, CSMD3, NPL2, KIAA1539, TOLLIP, COG2, RBM19, ADAMTS4, SLC8A2, PGR |
| hsa-miR-3911 | 26 | MAP2, LPP, FGF11, ANK3, ZNF233, JMJD8, WBP2NL, C5orf41, FLJ25076, CPEB2, ZNF34, WHSC1L1, INO80D, BCOR, PAIP2, CYP2S1, C11orf41, HSPBP1, PLCB1, FUT9, CADPS, SSR1, SLCO2A1, RORA, RAD23B, MEIS2 |
| hsa-miR-4689 | 24 | IPO5, IGSF1, DRP2, ALDH1A3, FLJ44082, FLJ43859, C18orf34, RGAG4, TMEM136, KRT40, KLHL29, SNX27, ARID1B, HBP1, FNDC3A, ZNF652, BAZ2A, MED13, ZBTB39, LUZP1, SGK1, ROCK1, PCTK2, NRAS |
| hsa-miR-3196 | 35 | KAL1, JUND, IVD, HOXD13, H2AFX, FZD2, EEF1A2, NKX3-2, LGI3, U2AF1L4, FGD5, LRRC15, BBS5, CYP4F22, ZNF787, C16orf55, FOXP4, SCRT1, TRIM48, RCN3, MAN1C1, GATAD2A, TLX3, BBC3, ZBTB32, OTP, TSPAN9, CPLX1, INA, SYNGAP1, PPFIA3, DPF1, SLC39A7, PAX2, NFIX |
| hsa-miR-4429 | 182 | MCL1, MAP1B, LAMP2, KCNS3, IGF1R, AGFG1, HOXA5, HIVEP2, HIC1, GSPT1, GNAI1, GABPB1, ESRRG, EREG, DHX15, DCC, DBN1, DAG1, CUX1, CPD, CDK6, CDH2, RUNX1T1, MPPED2, KLF5, BMPR1A, ATRX, ATP6V1A, ZNF705D, METTL10, SPOPL, MSL1, PRTG, ANKRD52, NAP1L5, BOD1L, PAN3, FBXO33, BRWD3, SDK1, NRK, C14orf147, MIER3, FAM117B, HECTD2, CXorf39, RBM45, MSI2, DNER, MTDH, ABHD13, DPY30, LCOR, PCGF5, ING5, MEX3B, TMEM47, JHDM1D, ZFP91, KLHL15, FBXO11, NARG1, CSRNP3, SEMA6D, DHDDS, SHCBP1, KLHL36, TBL1XR1, TMEM108, WNK1, MRPS25, GMCL1, PAPD5, SMAP1, MIER1, FAM160B1, PCDH19, NLN, CNOT6, PAK7, PCDHA1, PCDHA2, PCDHA3, PCDHA4, PCDHA5, PCDHA6, PCDHA7, PCDHA10, PCDHA11, PCDHA12, PCDHA13, PCDHAC1, PCDHAC2, NXT2, LMO3, ENAH, YOD1, ARL8B, FAM70A, BANP, INO80D, RP11-35N6.1, TMEM106B, FAM63B, PLEKHA5, CHIC1, CMPK1, KLF13, AZIN1, FAM49B, WAC, GCNT4, CNOT7, USP25, CDH20, AFF4, AP3M1, COPG2, DAZAP1, NCAPD3, CAMSAP1L1, ATP11A, RAB18, CNKSR2, KIAA0831, ZNF652, SEC63, ZNF268, ARPP19, STAG2, IGF2BP3, IPO7, SYNCRIP, SEMA3A, CACNG2, PLXNC1, AKT3, HELZ, SMG7, RHOBTB1, N4BP1, CREB5, ONECUT2, SEP15, B4GALT6, COPS2, CYTH1, LRRFIP1, PCSK7, EXO1, INA, SGPL1, NRP1, TNKS, CDC2L5, CGGBP1, ULK1, XPO1, TSC1, NR2C2, TPM3, TFAP2B, TDG, TAF5, TROVE2, RIT1, RASA1, RAP1A, TWF1, PTEN, MAPK1, PPM1B, PNN, PLS1, PHF1, PBX3, PBX1, NPAS2, MN1, MMP16, MLLT3, KITLG |
| hsa-miR-4327 | 131 | MAGEA6, MAGEA3, IPO5, IGF1R, HPGD, CXCL3, FMR1, FLNB, MLANA, GPC5, ERCC4, DLG1, CREBBP, CDC6, CBFB, CALCR, KLF9, BAAT, ATP2B1, ARSB, ADPRH, ADCYAP1, ABAT, TMEM41B, HAPLN4, KRTAP26-1, DKFZp686E2433, ZNF677, CLEC4D, FAM126B, MYO1H, UBN2, ZBTB38, ATP6V0D2, SLC16A9, CCNY, FAM18B2, FAM76A, MDGA2, C9orf66, CMTM4, FBXL14, ZNF440, C16orf46, NAT12, ARAP2, TMEM200A, ZNF439, ZNF700, ZC3H12C, PLA2G12B, SLC12A8, C3orf26, SLC37A3, FBXO30, VANGL1, DOCK8, C5orf44, FAM57A, ARHGAP28, SAP30L, DCTPP1, PRDM15, NEUROG2, CHP2, USP31, SHROOM4, GPR126, NIPAL3, PCNP, SLC17A6, UGCGL1, EIF5A2, KIAA1370, PCDHAC2, MLL5, HDAC8, ZNF415, ENAH, FAM46A, SLC29A3, CDKAL1, SUFU, PEX5L, FAM13B, NUSAP1, FAM18B, MYEF2, CLEC2D, ATAD2, FILIP1, RGS17, ARL5A, CLEC5A, DDAH1, R3HDM1, ISCU, PHF15, KIAA0802, UBXN4, LARP5, TNRC6B, ERP44, FOXJ3, FNDC3A, CPSF6, SPIN1, FGL2, ENOX2, ABCC4, TSHZ1, YAF2, ARHGAP25, SV2B, PUM1, ABCG1, BAG5, ZNF264, NAPG, ADAM20, VAMP4, ZNF20, SYT4, SYN2, SOX2, SMARCD2, SLC20A1, SALL1, ROBO2, PKN2, YBX1 |
| hsa-miR-150-3p | 115 | SMAD5, LMO7, LAMP2, IPO5, IGF1, HMGB3, HK1, FOXO3, ESRRG, MEGF9, DYRK1A, DUSP7, CYP1B1, CYBB, CAV2, CENPP, C2orf68, TMEM179B, ANKDD1A, NANOS1, ACER2, GADL1, SFRS12IP1, JAKMIP3, TMEM9, DHX36, C1orf71, GBP6, ZDHHC15, RLBP1L1, PRELID2, SLC30A7, RTP1, WDR17, SLC2A13, MYOCD, CACNA2D4, OXNAD1, FMNL3, DIXDC1, THAP2, ARHGAP24, RNF170, DOCK8, NUBPL, SVEP1, MOBKL2B, QTRTD1, SH3TC2, RAPH1, NOM1, ABCG8, ZBTB4, KIAA1486, CRAMP1L, KIAA1377, DENND4C, RFK, PCMTD2, MRPS10, FAM46C, A2BP1, SGTB, XRN1, SLC38A2, PPME1, ZDHHC2, PI15, DUOX2, ACAD9, EHF, SERBP1, IFIT5, ACSL6, FBXW11, CEP68, RTF1, SNX13, MGAT4A, AP1GBP1, TLK2, ARPP-21, HBS1L, GNA13, PDLIM5, NEBL, ZBTB33, AMMECR1, OXSR1, ZBTB24, ARHGEF2, MTMR4, RNF8, ADAM12, ZMYM2, ZNF708, TMF1, TBCD, STRN, SPOCK1, SLC5A3, SC5DL, RPS23, RAB3B, PRKAA2, PPP2R3A, PLAGL1, PAPPA, PAFAH1B1, NOTCH3, NEFL, MYO6, MYD88, MYB, NR3C2 |
| hsa-miR-642b-3p | 333 | LPP, KCNJ8, ITGB8, IL11, IFNA8, TNC, HNRNPU, CXCL3, GPR34, LPAR4, GOLGA1, GNAI1, GCLM, GFPT1, GABRB3, GAB1, FRK, FLT1, FOXF2, EIF2B1, EHHADH, MEGF9, CYLD, CSTF3, CREM, COPA, FOXN3, CTSC, SCARB2, CD1B, CCNA2, CCK, CBL, CASP10, CALM1, C7, ARSB, ANGPT1, AMHR2, AMBN, AK3L1, AHR, ADRBK2, ADORA2B, ADARB2, ACVR2B, LOC100130451, C16orf52, D2HGDH, CLLU1, FLJ41562, C1QL3, USP27X, VGLL3, MIA3, C15orf38, MACC1, C2orf55, KCNT2, SLC6A19, CCDC144NL, EIF4E3, C9orf126, SCARA5, RIMKLA, PRTG, TAS2R20, GCET2, EPGN, SPESP1, LNX2, RSBN1L, GPR115, FAM13C, DOK6, KIAA2018, ANO5, ZNF449, DNAJC18, EIF2C4, EIF2C3, ZNF675, LONRF2, CAMSAP1, RNF145, CMTM8, C3orf59, TTC14, ZNF555, DPY19L3, ZNF417, GCOM1, TRUB1, C20orf112, FUNDC1, SLITRK4, ABRA, TMEM139, CD109, NCOA7, FLJ25076, CD200R1, AP1S3, NIPA1, LRIG3, CYYR1, NUS1, ZNF257, WDR67, TMEM169, ANKRD44, KLHL13, GTPBP10, RERG, C9orf3, ATAD1, ZNF347, C2orf88, ARL6, DNAL1, EPPK1, TSPAN14, NIPA2, GRINL1A, APOL6, C2orf37, WDR32, ELOVL6, KCTD15, RASL11B, ZNF649, TMEM135, AGXT2L1, PAPD5, ELTD1, C10orf84, MID1IP1, ZNF529, POGK, SH3RF1, KIAA1407, KLHL14, SERINC1, MTUS1, MCOLN1, CD177, C12orf4, KTELC1, C5orf15, C11orf30, FEM1C, C8orf4, BEX4, LANCL2, DHX32, NUP133, PARVA, ZCCHC8, FAM48A, TRIM36, SYNJ2BP, SPTLC3, RBM41, MSL2, GPATCH2, PAK1IP1, INO80D, BCOR, FNBP1L, SLC35F2, ING3, CCDC93, PCDH18, GFOD1, CHRAC1, SYT17, LSM8, ASB2, GULP1, C4orf18, GLRX5, SAR1B, FAM108B1, TRNT1, FAM135B, ST8SIA3, ZNF295, NXPH1, FLVCR1, SERP1, NAAA, KIAA1274, ANKRD1, RNF11, PHF19, WIPI2, ERC2, LRIG1, L3MBTL, MTCH1, BHMT2, TSPAN12, TARDBP, ASTN2, RRP12, JMJD6, SYT11, GARNL4, FSTL4, PHLPPL, DIP2C, EFR3B, TRAK1, IKZF2, PDCD10, RABL2A, RABL2B, FGFR1OP, STMN2, ABHD2, NUDT21, GPR75, PPARGC1A, FAM12A, CPLX2, ZNF268, NFAT5, CUGBP2, EXOC5, PDPN, STAMBP, PDLIM5, SYNCRIP, IKZF1, ZNF267, GPR64, HIPK3, HCN4, ABI1, DCLRE1A, ZBTB24, GINS1, BMS1, BCLAF1, KIAA0195, KIAA0513, USP6NL, CEP57, QKI, TJP2, NRXN2, RAB28, NDST3, CNOT8, GTF3C4, COPS2, ITGB1BP1, CCPG1, LRRFIP1, RABEP1, SPAG9, MPZL1, CDK5R1, IQGAP1, B3GALT1, USO1, CGGBP1, COPS3, PIK3R3, SUPT3H, CUL2, STK24, FZD1, PABPN1, NR4A3, REEP5, ZNF226, ZNF192, WT1, UGDH, TUFT1, TPMT, TMPO, TMOD1, TSPAN7, TIA1, TFRC, ZEB1, SYK, SLC5A3, SKP1, SFRS1, SFRP4, CXCL5, SCN3A, RPE, RB1, RAD51L1, PEX19, PTPRR, PTPRO, PTPRG, PTGER3, PTEN, PROX1, MAP2K6, PRKACB, PRIM1, PPP1R3D, PPM1A, PKP2, PKNOX1, PGR, PFTK1, PFKFB2, ATP8B1, PEPD, PDE4D, PCDH7, PAK2, OPCML, OPA1, NNAT, NFYB, NFIA, NDUFA4, CEACAM6, MYO5A, MTF1, MMP10, MLLT4, ATXN3, MEIS2 |
| hsa-miR-1249 | 9 | HOXB8, GRB14, TBPL2, RNF149, MOBKL1A, ING3, PHLDA1, GPR64, NPY1R |
| hsa-miR-3195 | 9 | GRIK5, GNG7, TLX3, BBC3, TBX1, ST3GAL2, POU3F1, PITX1, NFIX |
| hsa-miR-5195-3p | 183 | SMAD3, LOX, TNPO1, KCNA6, ITGB8, H2AFX, GPD2, GGT7, FLT1, FLI1, ACSL4, ELK4, EBF1, DUSP6, DAB2, CSTF3, AP3S1, CBFB, CAPZB, CACNA1D, C11orf9, KLF5, BNIP3, BMP3, ARF6, ANGPT2, AP1G1, ADD3, ACTG1, ACTB, ABCA1, ZNF704, SAMD12, NSUN4, GJB7, MTX3, NEGR1, SERINC5, UBN2, EBF3, IPMK, ATXN7L1, ANO6, GLIS3, PAPD4, ITPRIPL2, DENND5B, SMCR8, ZBTB46, TMEM178, VASN, OSBPL1A, LENG8, SLC25A25, SPSB4, YTHDC1, SELI, SSH2, UNC119B, KDM2B, FAM126A, SLITRK6, DNAL1, ARHGAP24, ADPGK, SBF2, RNF170, SNX27, UXS1, ALS2CR8, C15orf29, PARP8, ZBTB10, SPATS2, GPBP1, USP46, FNDC4, FNDC3B, ACBD3, MPP5, BACH2, SENP2, C6orf115, FAM108C1, ZSWIM6, CACHD1, EPB41L5, PHRF1, FAM135A, SEMA6A, TAOK1, NUFIP2, SRGAP1, MKL2, USP31, PAK7, CTNNBIP1, MBNL3, KIF21A, WDR33, UBA6, FAM70A, KLHL28, MBTD1, INO80, RNF216, XRN1, RIN2, YTHDF2, AMOTL2, PLCE1, ZDHHC9, PHF20L1, FAM108B1, SOCS7, HIPK2, PDCD4, ZFYVE26, ATP1B4, SRGAP2, NEDD4L, TRIM2, KLHL18, ANKRD28, TBC1D12, PLCL2, LARP5, HIC2, FNDC3A, GABARAPL2, CPSF6, C11orf58, FRS2, IVNS1ABP, SEMA3A, CITED2, NET1, GPHN, MPZL2, AKAP9, SCAMP3, AKT3, MED13, NUAK1, ZC3H11A, ELMO1, CLINT1, SH3PXD2A, AKAP12, ONECUT2, CABP1, MAP4K4, QKI, ZFYVE9, DLGAP1, VAMP4, SPOP, PTP4A2, YES1, TGFBR2, SOX11, SOX9, FSCN1, SLC1A2, ATXN2, RTKN, RREB1, RGS7, REV3L, RASA1, PXN, PTGFR, PRKX, PPP3CA, PFTK1, PCBP2, TNFRSF11B, NEDD9, NDUFA4, MYO6, MYO5A, MMP16, MEST |
| hsa-miR-92a-3p | 209 | MARK1, MAN2A1, SMAD7, KIF5B, ITPR1, ITGAV, ITGA5, INSIG1, HOXB8, GRIA3, GOLGA4, GOLGA3, GATA2, GAP43, FMR1, FHL2, FBN1, DUSP5, DSC2, DMXL1, DDX3X, COL12A1, COL1A2, LYST, CDKN1C, CDH10, CD69, CCNC, BCAT2, ATRX, ATP2A2, ADM, ADCY3, NKX2-4, FAM19A1, LIN28B, PTAR1, MIA3, RBPMS2, C9orf150, KSR2, SPRYD4, TMEM188, PIKFYVE, ZFC3H1, MIER3, GPR180, NKX2-3, CNTN4, RNF38, SESN3, CBLN4, SLC32A1, JMY, PCMTD1, CSMD3, FNIP1, TSGA14, ADAMTSL1, ZNF804A, SGK493, FAM110B, RHPN2, BTBD12, DNAJC30, YIPF4, KIAA1109, PCDH11Y, C6orf62, ANP32E, SGPP1, QSER1, TBL1XR1, PPCS, CCNJL, C17orf39, AIDA, FNDC3B, HERPUD2, MOAP1, GPBP1L1, PLEKHA1, PRDM13, KIAA1632, USP28, DPP10, BAHCC1, KLHL14, ARRDC3, ARID1B, KIAA1211, ZNF287, ADAMTSL3, SLC17A6, TULP4, WRNIP1, IQWD1, CDCA7L, FBXW7, UBE2W, SETD5, SLC25A36, ARMC1, BSDC1, C10orf118, DUS2L, DNAJB12, RSBN1, RBM47, ANKIB1, SLC38A2, C21orf91, BCL11A, RAB23, NLK, VPS54, POLK, ZDHHC3, ERGIC2, RNF141, NOX4, BAZ2B, GRHL1, MYLIP, PCDH11X, NPTN, PCOLCE2, LATS2, APPL1, NECAP1, TMEM87A, CD2AP, MORC3, DOCK9, SNX13, CIC, MYCBP2, PDZD2, PHLPPL, WDFY3, CPEB3, RNF44, DUSP10, POLS, TOB2, TACC2, KLF2, LHFPL2, TOB1, FRY, HIPK3, JOSD1, G3BP2, ZEB2, EDEM1, KIAA0430, SOCS5, SH3PXD2A, KLF4, MFHAS1, DLGAP2, TMSB4Y, WASL, CACNA1I, SYNJ1, KAT2B, DYRK2, SNN, FXR1, BTG2, UGP2, TSC1, TRIO, TRAF3, TGIF1, TEF, TEAD1, DYNLT3, TCF21, HNF1B, SYN2, SSFA2, SRPR, SOX4, MAP2K4, RPL15, ROBO2, RNF4, RGS3, REV3L, RAP1B, RAD21, PTPRO, PTGER4, PTEN, PAX9, PAFAH1B1, CLDN11, NSF, NOVA1, NFIA, NEFL, NEFH, NEFM, PPP1R12A, MYO5A, MTF1, ATXN3, DNAJB9 |
| hsa-miR-1273g-3p | 74 | KCNA7, AGFG1, HNRNPU, ELK4, SLC31A1, COL12A1, CNR1, CDK6, PLEKHG7, VGLL3, SH2D4B, ARID2, SYNPO2, ZNF497, C8orf45, AMZ1, SLFN13, SMCR8, C18orf19, UBE2Q2, SESTD1, COX19, DCTN5, MEGF10, SLC7A6OS, VANGL1, C1orf21, OBFC1, METTL8, SH3TC2, CHCHD7, TMEM185B, WNK1, C6orf106, XPNPEP3, GALNT11, PKNOX2, ALX4, ZNF490, TBC1D24, VANGL2, ALG1, HDAC8, ENAH, CDV3, FAM82A2, PHIP, TRIM44, PLEKHA5, KCNK10, TFCP2L1, FBXW2, RCOR1, KIAA0907, NFAT5, POLD3, SPRY3, LPGAT1, SLC35E2, ZNF592, CFLAR, FGF23, FZD3, LRP8, UNG, MED22, SPN, MAPK1, NOS1, NFIA, PPP1R12B, MMP13, KITLG, MDM4 |

**Supplementary Table 2.** All of the significantly enriched pathways for the target genes of differentially expressed miRNAs

| Pathway_ID | Name | Count | Target Genes | FDR |
| --- | --- | --- | --- | --- |
| hsa04740 | Olfactory transduction | 5 | CALM1, ADCY3, PRKX, PRKACB, ADRBK2 | 0.001989049 |
| hsa05205 | Proteoglycans in cancer | 31 | ACTG1, NRAS, PDCD4, ANK3, FZD1, ITGAV, ITGA5, IGF1, FRS2, CAV2, FLNB, CBL, PXN, FZD2, PPP1R12A, PIK3R3, MAPK1, PLCE1, GAB1, ITPR1, AKT3, ACTB, IGF1R, PPP1R12B, FGF23, PRKX, FGF11, IQGAP1, FZD3, PRKACB, ROCK1 | 0.004268857 |
| hsa04010 | MAPK signaling pathway | 32 | NRAS, JUND, PPM1A, CACNA1D, RAP1B, DUSP6, FLNB, DUSP5, MAPK1, CACNG2, MAP4K4, PPP3CA, AKT3, NLK, DUSP10, PTPRR, PAK2, FGF23, DUSP7, PRKX, CACNA2D4, TGFBR2, FGF11, RASA1, MAP2K4, ELK4, PPM1B, TAOK1, RAP1A, PRKACB, CACNA1I, MAP2K6 | 0.016391713 |
| hsa05414 | Dilated cardiomyopathy | 15 | ACTG1, ITGB8, ITGAV, DAG1, ITGA5, IGF1, CACNA1D, ATP2A2, CACNG2, ADCY3, ACTB, PRKX, CACNA2D4, PRKACB, TPM3 | 0.02136154 |
| hsa04015 | Rap1 signaling pathway | 27 | ACTG1, NRAS, ANGPT2, ADORA2B, CNR1, MLLT4, FLT1, IGF1, GNAI1, CALM1, RAP1B, ANGPT1, PIK3R3, MAPK1, PLCE1, LPAR4, AKT3, ADCY3, ACTB, IGF1R, FGF23, KITLG, FGF11, PLCB1, ARAP2, RAP1A, MAP2K6 | 0.018406673 |
| hsa04520 | Adherens junction | 13 | ACTG1, CREBBP, MLLT4, YES1, LMO7, WASL, MAPK1, NLK, ACTB, IGF1R, SMAD3, TGFBR2, IQGAP1 | 0.016409495 |
| hsa05412 | Arrhythmogenic right ventricular cardiomyopathy (ARVC) | 13 | ACTG1, CDH2, ITGB8, ITGAV, DAG1, ITGA5, DSC2, CACNA1D, PKP2, ATP2A2, CACNG2, ACTB, CACNA2D4 | 0.016093657 |
| hsa04724 | Glutamatergic synapse | 17 | GRIK5, GNAI1, CACNA1D, SLC1A2, MAPK1, ITPR1, PPP3CA, ADCY3, SLC17A6, DLGAP1, GNG7, PRKX, PLCB1, GRIA3, PRKACB, ADRBK2, SLC38A2 | 0.022324586 |
| hsa04360 | Axon guidance | 18 | NRAS, DCC, SEMA6D, SRGAP1, SEMA3A, SEMA6A, PAK7, GNAI1, NRP1, MAPK1, ROBO2, PPP3CA, PAK2, RGS3, RASA1, SRGAP2, PLXNC1, ROCK1 | 0.02165145 |
| hsa04014 | Ras signaling pathway | 27 | NRAS, ANGPT2, MLLT4, FLT1, PAK7, IGF1, CALM1, RAP1B, ANGPT1, PIK3R3, MAPK1, PLCE1, ARF6, GAB1, AKT3, IGF1R, PAK2, FGF23, GNG7, PRKX, KITLG, FGF11, SYNGAP1, RASA1, RAP1A, PRKACB, PLA2G12B | 0.024181237 |
| hsa04510 | Focal adhesion | 25 | ACTG1,COL1A2,ITGB8,ITGAV,FLT1,PAK7,ITGA5,IGF1,PARVA,CAV2,RAP1B,FLNB,PXN,PPP1R12A,PIK3R3,MAPK1,AKT3,ACTB,IGF1R,PAK2,PPP1R12B,TNC,PTEN,RAP1A,ROCK1, | 0.024249327 |
| hsa05211 | Renal cell carcinoma | 11 | CREBBP,NRAS,PAK7,RAP1B,PIK3R3,MAPK1,GAB1,AKT3,PAK2,RAP1A,CUL2, | 0.034959326 |
| hsa05410 | Hypertrophic cardiomyopathy (HCM) | 13 | ACTG1,ITGB8,ITGAV,DAG1,ITGA5,IGF1,CACNA1D,ATP2A2,CACNG2,ACTB,PRKAA2,CACNA2D4,TPM3, | 0.03272926 |
| hsa04720 | Long-term potentiation | 11 | CREBBP,NRAS,CALM1,RAP1B,MAPK1,ITPR1,PPP3CA,PRKX,PLCB1,RAP1A,PRKACB, | 0.033980828 |
| hsa04261 | Adrenergic signaling in cardiomyocytes | 19 | CREM,ATP2B1,GNAI1,CACNA1D,CALM1,ATP1B4,PIK3R3,MAPK1,ATP2A2,CACNG2,CREB5,AKT3,ADCY3,PPP2R3A,PRKX,CACNA2D4,PLCB1,PRKACB,TPM3, | 0.034372729 |
| hsa04310 | Wnt signaling pathway | 18 | CREBBP,FZD1,SKP1,CTNNBIP1,VANGL2,VANGL1,FZD2,TBL1XR1,SENP2,PPP3CA,SFRP4,NLK,SMAD3,PRKX,PLCB1,FZD3,FBXW11,PRKACB, | 0.034284134 |
| hsa04730 | Long-term depression | 10 | NRAS,IGF1,GNAI1,NOS1,MAPK1,ITPR1,GNA13,IGF1R,PLCB1,GRIA3, | 0.036973336 |
| hsa04350 | TGF-beta signaling pathway | 12 | CREBBP,SKP1,SMAD7,ZFYVE9,BMPR1A,AMHR2,MAPK1,SMAD3,SMAD5,ACVR2B,TGFBR2,ROCK1, | 0.039505744 |
| hsa04727 | GABAergic synapse | 13 | GABARAPL2,SLC32A1,GNAI1,CACNA1D,GABRB3,GPHN,ADCY3,GNG7,PRKX,NSF,ABAT,PRKACB,SLC38A2, | 0.037803999 |
| hsa05218 | Melanoma | 11 | NRAS,IGF1,PIK3R3,MAPK1,CDK6,AKT3,RB1,IGF1R,FGF23,FGF11,PTEN, | 0.038343112 |
| hsa03015 | mRNA surveillance pathway | 13 | NUDT21,NXT2,HBS1L,CSTF3,PABPN1,SMG7,MSI2,WDR33,CPSF6,DAZAP1,PPP2R3A,GSPT1,PNN, | 0.037781217 |
| hsa04068 | FoxO signaling pathway | 17 | CREBBP,NRAS,GABARAPL2,SGK1,IGF1,PIK3R3,MAPK1,AKT3,NLK,PRKAA2,IGF1R,BNIP3,SMAD3,KLF2,TGFBR2,FOXO3,PTEN, | 0.036456906 |
| hsa04723 | Retrograde endocannabinoid signaling | 14 | SLC32A1,CNR1,GNAI1,CACNA1D,GABRB3,MAPK1,ITPR1,ADCY3,SLC17A6,GNG7,PRKX,PLCB1,GRIA3,PRKACB, | 0.040707714 |
| hsa03018 | RNA degradation | 11 | CNOT8,POLS,TOB1,LSM8,PAN3,CNOT6,TOB2,XRN1,BTG2,CNOT7,DHX36, | 0.039990432 |
| hsa05214 | Glioma | 10 | NRAS,IGF1,CALM1,PIK3R3,MAPK1,CDK6,AKT3,RB1,IGF1R,PTEN, | 0.046277386 |
| hsa04066 | HIF-1 signaling pathway | 14 | CREBBP,ANGPT2,PFKFB2,FLT1,IGF1,ANGPT1,PIK3R3,MAPK1,CYBB,AKT3,HK1,IGF1R,TFRC,CUL2, | 0.046913481 |
| hsa05100 | Bacterial invasion of epithelial cells | 11 | ACTG1,ITGA5,CD2AP,CAV2,CBL,PXN,PIK3R3,WASL,ELMO1,GAB1,ACTB, | 0.048958993 |
| hsa04960 | Aldosterone-regulated sodium reabsorption | 7 | SGK1,IGF1,ATP1B4,PIK3R3,MAPK1,NEDD4L,NR3C2, | 0.04971556 |
| hsa04713 | Circadian entrainment | 13 | GNAI1,CACNA1D,CALM1,NOS1,MAPK1,ITPR1,ADCY3,GNG7,PRKX,PLCB1,GRIA3,PRKACB,CACNA1I, | 0.048056008 |
